# Concordance of the ACR TI-RADS Classification With Bethesda Scoring and Histopathology Risk Stratification of Thyroid Nodules

**DOI:** 10.1001/jamanetworkopen.2023.31612

**Published:** 2023-09-13

**Authors:** Elaine Y. F. Huang, Nern Hoong Kao, Snow Yunni Lin, Isabelle J. H. Jang, Kimberley Liqin Kiong, Anna See, Nanda Venkatanarasimha, Kristen Alexa Lee, Chwee Ming Lim

**Affiliations:** 1 Department of Otorhinolaryngology, Head and Neck Surgery, Singapore General Hospital, Singapore; 2 Department of General Surgery–Head and Neck Surgery, Changi General Hospital, Singapore; 3 Yong Loo Lin School of Medicine, National University of Singapore, Singapore; 4 Surgery Academic Program, Duke-NUS Medical School, Singapore; 5 Department of Diagnostic and Interventional Radiology, Singapore General Hospital, Singapore

## Abstract

**Importance:**

Although most thyroid nodules are benign, 10% to 15% of them harbor cancer. Thyroid ultrasonography is useful for risk stratification of nodules, and American College of Radiology Thyroid Imaging Reporting and Data System (ACR TI-RADS) classification provides recommendations for fine-needle aspiration cytology (FNAC) based on objective ultrasonographic features of these nodules.

**Objective:**

To validate the concordance of ACR TI-RADS classification with Bethesda classification and histopathology.

**Design, Setting, and Participants:**

This retrospective cohort study was performed to evaluate the concordance of ACR TI-RADS classification with Bethesda classification and histopathology and was conducted in Singapore General Hospital Outpatient Otolaryngology clinic in March 2021 to May 2021. Data analysis was performed in May 2021.

**Main Outcomes and Measures:**

Results were deemed concordant when ACR TI-RADS recommendations aligned with Bethesda scores. Conversely, results were classified as nonconcordant with Bethesda scores and/or histopathology results when nodules that were recommended for FNAC yielded benign results or nodules that were not recommended for FNAC yielded malignant results.

**Results:**

A total of 446 patients (370 women [83%]; mean [range] age, 60 [24-89] years) who underwent ultrasonography of the thyroid and ultrasonography-guided thyroid FNACs were identified. A total of 492 of 630 nodules (78.1%) were benign on FNAC (Bethesda II). Score 3 ACR TI-RADS nodules yielded the highest negative predictive values: 94.6% (95% CI, 92.9%-95.9%; *P* < .001) compared with Bethesda scoring and 100.0% (95% CI, 15.8%-100.0%; *P* = .003) compared with histopathology. Score 4 or 5 ACR TI-RADS nodules yielded positive predictive values of 2.8% and 16.2%, respectively, compared with Bethesda scoring and 6.1% and 66.7%, respectively, compared with histopathology. Small (<1.5 cm) ACR TI-RADS nodules of scores of 4 and 5 that were not recommended for FNAC yielded a malignant risk of 5.7% and 25.0% on Bethesda 5 and 6, respectively. On surgical excision, 5 of 46 (10.9%) ACR TI-RADS 4 nodules and 15 of 21 (71.4%) of ACR TI-RADS 5 nodules were confirmed to be malignant. Among nodules initially not recommended for FNAC, histopathology-proven cancer was found in 4 of 13 (30.7%) and 3 of 6 (50.0%) of nodules, respectively.

**Conclusions and Relevance:**

These findings suggest that ACR TI-RADS score 3 nodules have a low risk of cancer and should be considered for FNAC only if nodules are 2.5 cm or larger. Patients with small (<1.5 cm) ACR TI-RADS 4 and 5 nodules should be appropriately counseled for FNAC to exclude cancer.

## Key Points

**Question:**

Are American College of Radiology Thyroid Imaging Reporting and Data System (ACR TI-RADS) recommendations concordant with Bethesda scoring and histopathology?

**Findings:**

In this cohort study of 446 patients with 630 thyroid nodules, ACR TI-RADS score 3 nodules had high concordance and negative predictive values (94.6% and 100.0%, respectively) with Bethesda scores and histopathology; the ACR TI-RADS nodules with a score of 4 or 5 showed low positive predictive values (2.8% and 16.2%, respectively) with Bethesda scores but higher positive predictive values (6.1% and 66.7%, respectively) with histopathology. ACR TI-RADS 4 or 5 nodules had cancer rates of 10.9% and 71.4%, respectively, after thyroidectomies.

**Meaning:**

These findings suggest that across ACR TI-RADS scores, ACR TI-RADS 3 is most useful to select benign thyroid nodules not requiring fine-needle aspiration cytology.

## Teaser

This cohort study examines the concordance of American College of Radiology Thyroid Imaging Reporting and Data System (ACR-TIRADS) classification with Bethesda classification and histopathology for thyroid nodules.

## Introduction

Thyroid nodules are defined by American Thyroid Association as “discrete lesions within the thyroid gland, radiologically distinct from surrounding thyroid parenchyma.”^1^ Most thyroid nodules are benign, with only 10% to 15% harboring cancer.^1^ The annual worldwide incidence rate of thyroid cancer was reported to be approximately 5.4% in men and 6.5% in women, making thyroid cancer the fifth most common cancer diagnosed in women and the most common cancer in women younger than 25 years.^2^ The first line of investigation for thyroid nodules is high-resolution ultrasonography.^3^ Nodules that are sonographically suspicious for cancer are subjected to fine-needle aspiration for cytology (FNAC), during which they are further stratified according to the Bethesda risk stratification cytological classification first developed in 2007.^4^

The American College of Radiology–Thyroid Imaging Reporting and Data System (ACR TI-RADS) risk stratifies thyroid nodules of cancer according to ultrasonologic features, such as composition, echogenicity, shape, margin, and echogenic foci.^5^ These nodules are scored on the basis of their respective features, allowing recommendations for the necessity of follow-up, frequency of surveillance, and need for FNAC. Fine-needle aspiration cytology is not recommended for ACR TI-RADS thyroid nodules with scores of 1 and 2, as these nodules do not display any worrisome features and are likely benign. Conversely, ACR TI-RADS nodules with scores of 3, 4, and 5 are recommended for either FNAC or ultrasonography surveillance depending on their size. For thyroid nodules that require FNAC, the cytology results are risk stratified according to the Bethesda classification, which identifies potentially malignant nodules for appropriate surgical intervention.^6,7^

As previously suggested by Tessler et al,^8^ ACR TI-RADS is beneficial in identifying benign nodules and has the potential to reduce unnecessary FNAC for benign nodules. Hence, this study aimed to validate the concordance of ACR TI-RADS classification with Bethesda classification and final histopathologic findings.

## Methods

This was a single-center, retrospective cohort study of adult patients older than 21 years who underwent ultrasonography of the thyroid and accompanying ultrasonography-guided (USG) FNAC of thyroid nodules. Correlative histopathology reports of patients who had undergone thyroidectomies were also collected.

Ultrasonography of the thyroid was performed by trained radiographers and independently reported by specialist radiologists at our institution. All radiology reports were performed using ACR TI-RADS classification, which facilitated consistent data accrual in this study. Patients who did not undergo thyroid ultrasonography at our center and patients who received previous radiotherapy to the head and neck region were excluded.

This study adhered to the Strengthening the Reporting of Observational Studies in Epidemiology (STROBE) reporting guideline checklist for reporting of cohort studies. Data were gathered via electronic health records. Ethical approval and consent exemption was obtained from the institutional review board of Singapore General Hospital before commencement of the study.

### Evaluation of Concordance Between ACR TI-RADS and Bethesda Classifications

For the purpose of the study, *concordance* was defined as when a nodule that was recommended for FNAC (according to ACR TI-RADS criteria) was found to be Bethesda classification 2 on FNAC or had a benign postoperative specimen. Concordance was also defined when a nodule that was recommended for FNAC (according to the ACR TI-RADS score) was found to be Bethesda classification 4 or 5 on FNAC or had malignant histopathology results. Bethesda classification 1, 3 and 4 nodules without formal histopathology were not included in the analysis for concordance.

### Nonconcordance

*Nonconcordance* was defined when the findings were contradictory to the previously described methods (eg, Bethesda 5/6 on FNAC or a malignant histopathology in nodules that were not recommended for FNAC according to the ACR TI-RADS criteria). These were subgrouped to either nonconcordance by score (not recommended for FNAC due to a low ACR TI-RADS score) or by size (not recommended for FNAC due to a small size that did not meet criteria for FNAC in respective ACR TI-RADS scores).

The subgroup analysis for ACR TI-RADS scores against the measured size on ultrasonography was performed. The nodule sizes were categorized according to American Joint Committee on Cancer 8th Edition TMN staging size for thyroid carcinoma (T1a, ≤1.0 cm; T1b, 1.1-2.0 cm; T2, 2.1-4.0 cm; and T3, >4.0 cm).^8^

### Statistical Analysis

Data analysis was conducted in May 2021 using SPSS statistical software version 21 (IBM). Sensitivity, specificity, positive predictive values (PPVs), and negative predictive values (NPVs) were calculated by comparing ACR TI-RADS recommendations for FNAC with cytology and ACR TI-RADS with histopathology. Results were analyzed by the Pearson χ^2^ test (2-sided) for categorical variables of benign or malignant nodules, with a significance level of *P* < .05.

## Results

A total of 446 patients who had undergone ultrasonography of the thyroid and USG thyroid nodule FNAC between August 1, 2018, to March 31, 2021, were identified. Most of the patients were female (370 women [83%]), whereas there were 76 male patients (17%). The age range of participants was 24 to 89 years old, with a mean age of 60 years. The study primarily included Chinese patients, accounting for 385 patients (86.3%); 24 patients (5.4%) were Indian, 17 (3.8%) were Malay, and 20 (4.5%) were of other ethnic groups. All data collection was conducted through electronic health records (Table 1).

**Table 1.** Demographic Characteristics

Characteristics	Patients, No. (%)
Sex	
Female	370 (83)
Male	76 (17)
Ethnicity	
Chinese	385 (86.3)
Indian	24 (5.4)
Malay	17 (3.8)
Other^a^	20 (4.5)
Age, mean (range), y	60 (24-89)
Age groups, y	
21-55	150 (33.6)
>55	296 (66.4)
Ultrasonography nodule size, mean (range), cm	2.5 (0.4-8.9)
American College of Radiology Thyroid Imaging Reporting and Data System score	
1 (Benign)	17 (2.0)
2 (Not suspicious)	22 (2.6)
3 (Mildly suspicious)	255 (30.3)
4 (Moderately suspicious)	429 (51.0)
5 (Highly suspicious)	119 (14.1)
Surgery	
Thyroidectomy	
Left hemithyroidectomy	14 (26.9)
Right hemithyroidectomy	20 (38.5)
Total thyroidectomy	17 (32.7)
Isthmusectomy	1 (1.9)
Neck dissection	
Unilateral central compartment only	1 (1.9)
Unilateral lateral neck only	0
Unilateral central compartment and lateral neck	4 (7.7)
Bilateral central compartment only	0
Bilateral lateral neck only	0
Bilateral central compartment and lateral neck	0
Histology^b^	
Benign nodules	64 (72.7)
Micropapillary thyroid carcinoma	5 (5.7)
Papillary thyroid carcinoma	15 (17.1)
Follicular thyroid carcinoma	3 (3.4)
Hurthle cell carcinoma	1 (1.1)

^a^ Data were gathered from electronic health record, and specific information of other ethnicities was not stated.

^b^ Further information on histology can be found in eTable 1 in Supplement 1.

### Distribution of Nodules

Thyroid ultrasonography was performed for 446 patients, with a total of 842 unique nodules identified and reported according to ACR TI-RADS. Of these nodules, 630 nodules had USG FNAC. eTable 1 in Supplement 1 summarizes the distribution of ACR TI-RADS 1 to 5 and Bethesda 1 to 5 scores of these nodules. Postoperative histopathology results of 88 nodules were available from 52 patients who underwent thyroid surgery after the initial investigations. Most of the nodules that underwent thyroid ultrasonography were scored ACR TI-RADS 4 (429 nodules [51.1%]), whereas ACR TI-RADS 1 (14 nodules [1.7%]) and 2 (22 nodules [2.6%]) nodules were the least frequently reported. Most of the FNAC results were Bethesda score 2 (492 of 630 nodules [78.1%]). No ACR TI-RADS 1, 2, or 3 nodules yielded a Bethesda 5 or 6 score on FNAC. Score 4 and 5 ACR TI-RADS nodules were more likely to be Bethesda scores 5 and 6: 2 of 347 (0.6%) and 6 of 347 (1.7%) ACR TI-RADS 4 nodules were Bethesda 5 and 6, respectively, whereas 3 of 101 (2.9%) and 12 of 101 (11.9%) ACR TI-RADS 5 nodules were Bethesda 5 and 6, respectively (eTable 1 in Supplement 1).

### Concordance Between ACR TI-RADS and Bethesda Scores

Of the 630 nodules that underwent FNAC, only 515 (81.7%) were included in the analysis for concordance of the ACR TI-RADS and the Bethesda scoring system. Nodules scored as Bethesda 1 (83 nodules), 3 (26 nodules), or 4 (6 nodules) were excluded from the analysis, because these Bethesda categories cannot be adequately classified as benign or malignant. According to the ACR TI-RADS recommendation, categories 1 and 2 are not recommended for FNAC. In these categories, all 12 nodules (5 ACR TI-RADS 1 and 7 ACR TI-RADS 2) that underwent FNAC yielded benign cytology results, supporting the ACR TI-RADS recommendation. Score 3 ACR TI-RADS nodules demonstrated the highest NPV (94.6%; 95% CI, 92.9%-95.9%; *P* < .001) compared with the FNAC results and 100% (95% CI, 15.8%-100.0%; *P* = .003) compared with histopathology. This means that 94.6% of ACR TI-RADS 3 nodules that were not recommended for FNAC were truly benign. However, ACR-TIRADS 5 had the highest PPV (16.2%) compared with other categories (the PPV was 2.8% for ACR-TIRADS 4). Score 4 or 5 ACR TI-RADS nodules had PPVs of 6.1% and 66.7%, respectively, compared with histopathology. Among all ACR TI-RADS categories, there were 95 nodules that were not recommended for FNAC but were biopsied because of patient preference. Among these nodules, 89 of 95 (93.7%) yielded concordant benign cytology (NPV, 92.1%; 95% CI, 86.4%-95.5%; *P* < .001). The remaining 6 nodules demonstrated malignant cytology results. These malignant nodules were not recommended for FNA according to the size criteria (<1.5 cm). Of these 6 nodules, 1 was classified as ACR TI-RADS 3, 2 were ACR TI-RADS 4, and 3 were ACR TI-RADS 5 (Table 2). Small (<1.5 cm) ACR TI-RADS 4 and 5 nodules that were not recommended for FNAC yielded a malignant risk of 5.7% and 25.0% on Bethesda 5 and 6, respectively. One of 8 subcentimeter ACR TI-RADS 5 nodules (12.5%) that were sent for FNAC because of patient preference had cytology-proven papillary thyroid carcinoma.

**Table 2.** ACR TI-RADS Concordance With Bethesda Scores^a^

Concordance with Bethesda score	ACR TI-RADS score											
	1		2		3		4		5		All	
	Yes	No	Yes	No	Yes	No	Yes	No	Yes	No	Yes	No
Concordance	0	5	0	7	0	35	7	33	11	9	18	89
Nonconcordance (due to score)	0	0	0	0	100	0	245	0	57	0	402	0
Nonconcordance (due to size)	0	0	0	0	0	1	0	2	0	3	0	6
Total	0	5	0	7	100	36	252	35	68	12	420	95
Sensitivity, %	NA		NA		0		70		78.9		69.2	
Specificity, %	NA		NA		25.9		11.9		13.6		18.8	
Positive predictive value, %	NA		NA		0		2.8		16.2		4.3	
Negative predictive value (95% CI)	NA		NA		94.6 (92.9-95.9)		91.7 (80.2-96.8)		75.0 (48.2-90.7)		92.1 (84.6-95.5)	
*P* value^b^	NA		NA		<.001		<.001		<.001		<.001	

Abbreviations: ACR TI-RADS, American College of Radiology Thyroid Imaging Reporting and Data System; NA, not applicable.

^a^ Results were analyzed by the Pearson χ^2^ test for categorical variables of benign or malignant nodules, with significance level set at *P* < .05.

^b^ Analysis for ACR TI-RADS 1 and 2 not performed (small number).

### Concordance Between ACR TI-RADS and Histopathology

The overall prevalence of histopathology-proven benign results in the study population was 75.0%. When comparing ACR TI-RADS with histopathology, 26 of 31 nodules (83.9%) that were not recommended for FNAC according to ACR TI-RADS were concordant with histopathology results. The remaining 5 nodules (16.1%) were nonconcordant according to ACR TI-RADS recommendations. These nodules were all benign on histopathology, giving rise to an NPV of 83.9% (95% CI, 69.8%-92.1%; *P* < .001). All ACR TI-RADS 1 and 2 nodules that were eventually surgically removed yielded concordant benign histopathology results (0 and 6 nodules, respectively). However, statistical analysis was not performed for these nodules owing to the small sample sizes of these scores (Table 3). On surgical excision, 2 of 15 ACR TI-RADS 3 nodules (13.3%) were proven to be follicular thyroid carcinoma following thyroidectomy. These 2 malignant nodules were classified as Bethesda 2 preoperatively and were large (>6 cm). Five of 46 ACR TI-RADS 4 nodules (10.9%) and 15 of 21 ACR TI-RADS 5 nodules (71.4%) were confirmed to be malignant (eTable 2 in Supplement 1).

**Table 3.** ACR TI-RADS Concordance With Histopathology^a^

ACR TI-RADS recommendation for FNA	ACR TI-RADS score											
	1		2		3		4		5		All	
	Yes	No	Yes	No	Yes	No	Yes	No	Yes	No	Yes	No
Concordance	0	0	0	6	2	6	2	9	10	5	14	26
Nonconcordance (due to size)	0	0	0	0	0	0	0	4	0	1	0	5
Nonconcordance (due to score)	0	0	0	0	7	0	31	0	5	0	43	0
Total	0	0	0	6	9	6	33	13	15	6	57	31
Sensitivity, %	NA		NA		100.0		33.3		90.9		73.7	
Specificity, %	NA		NA		46.2		22.5		50.0		37.7	
Positive predictive value, %	NA		NA		22.2		6.1		66.7		24.6	
Negative predictive value, % (95% CI)	NA		NA		100.0 (15.8-100.0)		69.2 (41.1-97.3)		83.3 (48.2-90.7)		83.9 (69.8-92.1)	
*P* value^b^	NA		NA		.003		<.001		.14		<.001	

Abbreviations: ACR TI-RADS, American College of Radiology Thyroid Imaging Reporting and Data System; FNA, fine-needle aspiration; NA, not applicable.

^a^ Analysis for ACR TI-RADS 1 and 2 not performed (small number).

^b^ Results were analyzed by the Pearson χ^2^ test for categorical variables of benign or malignant nodules, with significance level set at *P* < .05.

In this study, false-negative findings did occur, resulting in missing malignant nodules if clinicians applied the ACR TI-RADS recommendations strictly. Among the 45 ACR TI-RADS 4 nodules and 21 ACR TI-RADS 5 nodules that were surgically excised owing to patient preference, 13 ACR TI-RADS 4 nodules and 6 ACR TI-RADS 5 nodules were initially not recommended for FNAC. Of these nodules not recommended for FNAC, cancer was eventually proven in 4 of 13 (30.7%) and 3 of 6 nodules (50.0%), respectively. This resulted in the overall rates of malignancy for small nodules (<1.5 cm) not meeting size criteria for FNAC of 8.7% (4 of 46 nodules) for ACR TI-RADS 4 nodules and 14.3% (3 of 21 nodules) for ACR TI-RADS 5 nodules (eTable 2 in Supplement 1). These numbers should be interpreted cautiously given small sample sizes.

### Concordance Between Nodule Size and ACR TI-RADS

Nodules were also grouped according to size based on the American Joint Committee on Cancer 8th edition TMN staging (≤1.0 cm, 1.1-2.0 cm, 2.1-4.0 cm, or >4.0 cm) for subgroup analysis.^9^ The NPV of the ACR TI-RADS recommendation for FNAC was highest in the 2.1 to 4.0 cm group at 96.9% (95% CI, 83.1%-99.5%; *P* < .001) and lowest in the more than 4.0 cm group at 73.3% (95% CI, 57.9%-84.6%; *P* < .001). The NPVs were 88.9% (95% CI, 59.1%-97.8%; *P* < .001) for the 1.0 cm or less group and 95.6% (95% CI, 85.6%-98.7%; *P* < .001) for the 1.1 to 2.0 cm group. These results suggests that the ACR TI-RADS recommendations for or against FNAC are more accurate for nodules 1.1 cm to 4.0 cm (Table 4).

**Table 4.** Subgroup Size Analysis of ACR TI-RADS Concordance With Bethesda Scores

ACR TI-RADS recommendation for FNA	Ultrasonography size, cm							
	≤1		1.1-2.0		2.1-4.0		≥4.0	
	Yes	No	Yes	No	Yes	No	Yes	No
Concordance	1	20	11	48	6	17	0	4
Nonconcordance	5	3	123	3	205	1	68	0
Total	6	23	134	51	211	18	68	4
Sensitivity, %	66.7		81.8		85.7		20.0	
Specificity, %	22.9		20.5		16.4		18.0	
Positive predictive value, %	6.9		5.1		3.7		2.0	
Negative predictive value, % (95% CI)	88.9 (59.1-97.8)		95.6 (85.6-98.7)		96.9 (83.1-99.5)		73.3 (57.9-84.6)	
*P* value^a^	<.001		<.001		<.001		<.001	

Abbreviations: ACR TI-RADS, American College of Radiology Thyroid Imaging Reporting and Data System; FNA, fine-needle aspiration.

^a^ Results were analyzed by the Pearson χ^2^ test for categorical variables of benign or malignant nodules, with significance level set at *P* < .05.

## Discussion

Given that most thyroid nodules are benign, it is highly essential to select nodules for FNAC to prevent unnecessary invasive interventions. To that end, thyroid ultrasonography is currently the standard initial imaging investigation for thyroid nodules, and the ACR TI-RADS system was established in 2017 to assign a cancer risk stratification of nodules on the basis of ultrasonography features. This system also makes recommendations for subsequent management,^10^ with the overarching goal to reduce unnecessary FNACs or excessive surveillance of thyroid nodules.^11^

Our institution adopted the ACR TI-RADS reporting system to facilitate consistent radiology reporting of ultrasonography thyroid images. Beyond the risk stratification afforded by the ACR TI-RADS reporting system, other parameters, such as positive family history of thyroid cancer, history of head-neck irradiation, and patient preferences, were also considered when deciding on whether to proceed with thyroid FNAC. Surgery was recommended for Bethesda 4, 5, and 6 nodules and select Bethesda 3 nodules (after correlating with their corresponding thyroid ultrasonography findings and individual patient’s preferences). Among the Bethesda 2 nodules, surgery was performed when there were compressive symptoms from the nodules (typically among larger nodules >4 cm) or patient preference.

The current study demonstrated good concordance of ACR TI-RADS reporting system with Bethesda cytology among the 630 evaluable thyroid nodules. This finding was similar with previously reported series by Modi et al^12^ and Shaar et al^13^ among their series of 361 and 57 nodules, respectively. Collectively, ACR TI-RADS classification was useful in reducing number of unnecessary FNAC for benign nodules.^12,13^ When the ACR TI-RADS reporting system was correlated with eventual histopathology results, a stronger concordance of cancer than Bethesda cytology classification was found. To our knowledge, comparing ACR TI-RADS with histopathology validation has not been previously reported.

In previous studies,^12,14,15,16,17^ the concordance between ACR TI-RADS and Bethesda classifications was assessed by grouping TI-RADS scores into benign and malignant categories. For instance, authors considered ACR TI-RADS scores 1 to 3 benign, whereas ACR TI-RADS 4 and 5 were considered malignant. Concordance was determined when the sonographic benign group yielded benign Bethesda scores or when the sonographic malignant group yielded malignant Bethesda scores.^12,14,15,16,17^ However, our study took a different approach by assessing each ACR TI-RADS category independently in association with corresponding Bethesda and histopathology results. Our aim was to obtain a more comprehensive evaluation of each ACR TI-RADS category. Using this approach, we identified that nodules of the ACR TI-RADS 3 category demonstrated the highest NPV of 94.6% (95% CI, 92.9%-95.9%; *P* < .001), unlike an earlier study by Schenke et al,^17^ which found that ACR TI-RADS scores of 4 or greater had the highest NPV. This disparity is likely due to the differences in the methods of assigning concordance, as alluded to earlier.

Moreover, our findings suggest that clinicians should continue to exercise discretion when it comes to evaluating nodules at both extremes of sizes (≤1.0 cm and ≥4.0 cm). This is because nodules 1.1cm to 4.0 cm demonstrated the highest NPV compared with nodules of extremes of sizes of either 1.0 cm or less or 4.0 cm or greater. Although larger nodules are more likely to be recommended for FNAC, they may not always be associated with increased risk of cancer. None of the 72 nodules in our study that were 4.0 cm or larger yielded malignant FNAC results. This was consistent with previous studies that showed that large nodules larger than 4.0 cm were not associated with a higher risk of malignancy.^18,19^ For example, Tang et al^20^ reported that the false-negative rate of FNAC for 563 nodules 4.0 cm or greater was 6.6%, which was not statistically significantly different from the 4.2% false-negative rate for 4419 nodules smaller than 4.0 cm.

However, in our study, there were 2 of 15 ACR TI-RADS 3 nodules (13.3%) that were proven to be follicular thyroid carcinoma following thyroidectomy. These 2 malignant nodules were classified as Bethesda 2 preoperatively and were large (>6 cm). Although these ACR TI-RADS 3 nodules were initially considered nonconcordant compared with Bethesda classification, they were concordant with histopathology results. We postulate that this may be attributed to the interobserver variability of pathologists on reviewing the FNAC cytology, because diagnosing follicular thyroid cancer is fraught with challenges. Hence, management decisions for large thyroid nodules should be tailored to individual patients according to clinical, sonographic, and cytological features^14,20^ beyond ACR TI-RADS 3 classification alone.

Nodules with ACR TI-RADS 4 to 5 scores that are 1 cm or smaller are usually not recommended for FNA owing to the challenges in obtaining sufficient material on FNAC. This practical consideration is also consistent with the American Thyroid Association guidelines,^21^ which recommend that subcentimeter nodules should be considered for FNAC only when other risk factors of cancer are evident. In our study cohort, 1 of 8 subcentimeter ACR TI-RADS 5 nodules (12.5%) that were sent for FNAC because of patient preference had cytology-proven papillary thyroid carcinoma. However, with the growing practice of active surveillance for low-risk papillary thyroid microcarcinoma, it was noted that detecting these small cancers on FNAC may not be significantly associated with survival as these cancers are usually biologically indolent.^21^ In a large Japanese study by Miyauchi et al^22,23,24^ involving 1235 patients with micropapillary thyroid carcinoma over 10 years, only 8.0% of patients had size enlargement of more than 3 mm and 3.8% of patients had nodal metastasis. When these features were detected on active surveillance, surgical treatment following disease progression was not associated with the overall risk of subsequent recurrence or mortality.^22^

### Limitations

This study has limitations that should be mentioned. First, it was a single-institution study of a tertiary referral thyroid center, and the results cannot be directly generalized to other centers because of inherent differences in practices across various thyroid centers. Also, we were unable to perform subgroup analysis on ACR TI-RADS 1 and 2 thyroid nodules because of the small sample size in each group. Furthermore, our study excluded nodules that yielded indeterminate Bethesda scores of 1 (83 nodules), 3 (26 nodules), and 4 (6 nodules). Because these nodules were cytologically nondiagnostic and indeterminate, it was difficult to assign their concordance with ACR TI-RADS. Future studies with larger cohorts comparing ACR TI-RADS scores of Bethesda-indeterminate nodules with histopathology could provide valuable insights for clinical decision-making.

A total of 95 nodules in our study had FNAC performed despite not being recommended by ACR TI-RADS guidelines. The decision to proceed with FNAC was based on other factors, such as clinicians’ individual judgment based on nodule characteristics, patient risk factors of thyroid cancer, and individual patient’s preference. Although acknowledging this limitation, our findings demonstrated the utility of ACR TI-RADS in identifying for nodules not requiring FNAC in general. Conversely, 6 nodules initially not recommended for FNAC yielded malignant cytologic findings, emphasizing the importance of exercising caution and considering FNAC in cases in which other parameters beyond ACR TI-RADS are informative.

This study was retrospective and may have experienced selection bias due to missing data from patients lost to follow-up. This highlights the need for future prospective studies with comprehensive data collection and rigorous follow-up protocols to mitigate the effect of missing data.

In addition, owing to the operator-dependent nature of ultrasonography and FNA, interobserver variability in thyroid nodule risk stratification is possible. Nevertheless, the application of recognized classification systems aims to increase the reproducibility and reliability of ultrasonography and FNAC reporting across different centers and countries. Since their introduction, the ACR TI-RADS lexicon and Bethesda classification are widely accepted systems for risk stratification of thyroid nodules. Using these reporting system increases interobserver agreement by providing a clear scoring systems that standardizes thyroid nodule evaluation^25,26,27^ and facilitates uniform comparison of risk stratification of thyroid nodules across countries.

## Conclusions

The results of this retrospective cohort study suggest that ACR TI-RADS 3 thyroid nodules had a high NPV of 94.6% and carried a low risk of cancer. Sampling of these nodules should be considered only if they are 2.5 cm or larger to avoid missing a small percentage of thyroid cancer. Likewise, patients with small (<1.5 cm) ACR TI-RADS 4 and 5 nodules should be appropriately counseled for FNAC to exclude cancer. Our study confirms the potential use of the ACR TI-RADS system to guide clinicians toward appropriate selection of patients for FNAC and optimization of resources to manage this common clinical condition.

## Notes

Supplement 1. **eTable 1.** Summary of Distribution of Nodules Across Various ACR-TIRADS Categories **eTable 2.** Rate of Malignancy Across Individual Various ACR-TIRADS Categories

Supplement 2.
Data Sharing Statement
