# Supplementary material for: Concordance of the ACR TI-RADS Classification With Bethesda Scoring and Histopathology Risk Stratification of Thyroid Nodules
Source: JAMA Netw Open. 2023 Sep 13;6(9):e2331612. doi: 10.1001/jamanetworkopen.2023.31612 (PMC10500370; doi:10.1001/jamanetworkopen.2023.31612)
Supplement: Supplement 1. — eTable 1. Summary of Distribution of Nodules Across Various ACR-TIRADS Categories eTable 2. Rate of Malignancy Across Individual Various ACR-TIRADS Categories [file jamanetwopen-e2331612-s001.pdf]

Supplemental Online Content

Huang E, Kao NH, Lin SY, et al. Concordance of the ACR TI-RADS classification with Bethesda scoring and histopathology risk stratification of thyroid nodules. *JAMA Netw Open*. 2023;6(9):e2331612. doi:10.1001/jamanetworkopen.2023.31612

eTable 1. Summary of distribution of nodules across various ACR-TIRADS categories
eTable 2. Rate of malignancy across individual various ACR-TIRADS categories

This supplemental material has been provided by the authors to give readers additional information about their work.

© 2023 Huang E et al. *JAMA Network Open.*

eTable 1. Summary of distribution of nodules across various ACR-TIRADS categories

**ACR TI-RADS Categories**	**1**	**2**	**3**	**4**	**5**	**Total n (%)**
n (%)	14 (1.7)	22 (2.7)	255 (30.4)	429 (51.0)	119 (14.2)	842 (100)
**Subjected to FNAC** (%)	9 (1.4)	16 (2.5)	157 (25.0)	347 (55.1)	101 (16.0)	630 (100)
**BETHESDA** n (%)						
I	4 (44.4)	8 (50.0)	16 (10.2)	40 (11.5)	15 (14.9)	83 (13.2)
II	5 (55.6)	7 (43.7)	136 (86.6)	279 (80.4)	65 (64.4)	492 (78.1)
III	0 (0.0)	1 (6.3)	4 (2.6)	17 (4.9)	4 (3.9)	26 (4.1)
IV	0 (0.0)	0 (0.0)	1 (0.6)	3 (0.9)	2 (2.0)	6 (1.0)
V	0 (0.0)	0 (0.0)	0 (0.0)	2 (0.6)	3 (2.9)	5 (0.8)
VI	0 (0.0)	0 (0.0)	0 (0.0)	6 (1.7)	12 (11.9)	18 (2.8)
Total n,(%)	9 (100)	16 (100)	157 (100)	347 (100)	101 (100)	630 (100)
**Histopathology**						
Benign n (%)	0 (0.0)	6 (100)	13 (86.7)	41 (89.1)	6 (28.6)	66 (75.0)
Malignant n (%)	0 (0.0)	0 (0.0)	2 (13.3)	5 (10.9)	15 (71.4)	22 (25.0)
Total n (%)	0	6 (100)	15 (100)	46 (100)	21 (100)	88 (100)

© 2023 Huang E et al. *JAMA Network Open.*

eTable 2. Rate of Malignancy across individual various ACR-TIRADS categories

ACR TI-RADS categories (n)	Histology = n (nodules amongst these that did not meet size criteria for FNAC, if applicable)#						Rate of Malignancy (%)	Rate of malignancy of small nodules not meeting size criteria for FNAC (%)
	PTMC	PTC	FTC	HCC	Benign	**Total**		
1	0	0	0	0	0	**0**	0.0	0.0
2	0	0	0	0	6	**6**	0.0	0.0
3	0	0	2	0	13	**15**	13.3	0.0
4	2 (2)	3 (2)	0	0	41	**46**	10.9	8.7
5	2 (2)	11 (1)	1	1	6	**21**	71.4	14.3
**Total**	**4**	**15**	**3**	**1**	**66**	**88**		

PTMC = Papillary Thyroid MicroCarcinoma, PTC = Papillary Thyroid Carcinoma, FTC = Follicular Thyroid Carcinoma, HCTC = Hurthle Cell Carcinoma
#Nodules are recommended for FNAC if ACR TI-RADS 3 ≥2.5cm, ACR TI-RADS 4 ≥1.5cm, ACR TI-RADS 5 ≥1cm. Numbers in brackets represent number of nodules that did not meet size criteria for FNAC in their respective ACR TI-RADS categories, should there be any.

© 2023 Huang E et al. *JAMA Network Open.*
