# Supplementary material for: Concordance of the ACR TI-RADS Classification With Bethesda Scoring and Histopathology Risk Stratification of Thyroid Nodules
Source: JAMA Netw Open. 2023 Sep 13;6(9):e2331612. doi: 10.1001/jamanetworkopen.2023.31612 (PMC10500370; doi:10.1001/jamanetworkopen.2023.31612)
Supplement: Supplement 2. — Data Sharing Statement [file jamanetwopen-e2331612-s002.pdf]

Data Sharing Statement

Huang. Concordance of the ACR TI-RADS Classification With Bethesda Scoring and Histopathology Risk Stratification of Thyroid Nodules. *JAMA Netw Open*. Published September 06, 2023. doi:10.1001/jamanetworkopen.2023.31612

Data
Data available: Yes
Data types: Deidentified participant data
How to access data: gmslchm@nus.edu.sg
When available: With publication

Supporting Documents
Document types: None

Additional Information
Who can access the data: Researchers whose proposed use of data has been approved
Types of analyses: Combined analyses
Mechanisms of data availability: With signed agreement with institution
Any additional restrictions: no identifiers
